# A novel assay to measure calcification propensity: from laboratory to humans

**DOI:** 10.1038/s41598-020-74592-x

**Published:** 2020-10-16

**Authors:** M. Mar Perez, Miguel D. Ferrer, Marta Lazo-Rodriguez, Ana Zeralda Canals, Elisenda Banon-Maneus, Josep M. Campistol, Stephan Miller, Rekha Garg, Alex Gold, Carolina Salcedo, Joan Perelló

**Affiliations:** 1 Sanifit Therapeutics, Parc Bit - Europa Building, 2nd Floor, 07121 Palma de Mallorca, Spain; 2 Department of Fundamental Biology and Health Sciences, University of the Balearic Islands, Palma, Spain; 3 Laboratori Experimental de Nefrologia i Trasplantament (LENIT), Fundació Clínic per a la Recerca Biomèdica, Barcelona, Spain; 4 Spanish Kidney Research Network, ISCIII-RETIC REDinREN RD016/0 009, Madrid, Spain; 5 Sanifit Therapeutics, San Diego, CA USA; 6 Department of Medicine, Stanford University, Palo Alto, CA USA; 7 Laboratory of Renal Lithiasis Research, University of the Balearic Islands, Palma, Spain; 8 Present Address: PharmaDRS Consulting, LLC, San Diego, USA

**Keywords:** Calcification, Pharmacodynamics, Biomarkers

## Abstract

Cardiovascular calcification (CVC) contributes to morbidity and mortality in patients undergoing dialysis. We examined the pharmacodynamic effects of SNF472, a calcification inhibitor, on plasma calcium phosphate crystallization using spectrometric measurements, and its correlations with effects on CVC in rats or humans. Rats (N = 38) injected with vitamin D (days 1–3) to induce CVC were infused with saline or SNF472 (days 1–12). Inhibition of CVC was 50–65% with SNF472 3 mg/kg and ~ 80% with SNF472 10 or 30 mg/kg. SNF472 dose-dependently inhibited calcium phosphate crystallization, which correlated with inhibition of CVC (r = 0.628, *P* = 0.005). In patients with calciphylaxis (N = 14), infusion of SNF472 (~ 7 mg/kg) during hemodialysis for 12 weeks inhibited calcium phosphate crystallization by nearly 70%. In patients with CVC (N = 274), infusion of SNF472 during hemodialysis for 52 weeks inhibited calcium phosphate crystallization (placebo: 15%; 300 mg: 61%; 600 mg: 75%), which correlated with inhibition of CVC (r = 0.401, *P* = 0.003). These findings show a direct correlation between inhibition of calcium phosphate crystallization in plasma and inhibition of CVC both in a rat model and in humans, supporting the use of the pharmacodynamic assay in clinical trials as a potentially predictive tool to evaluate the activity of calcification inhibitors.

## Introduction

Cardiovascular calcification (CVC) is a major contributor to increased morbidity and mortality in patients undergoing dialysis^1^. CVC results in the deposition of calcium in vessel walls and the aortic valve, increasing the risk of cardiovascular events^2^. In calciphylaxis, an extreme form of CVC in small peripheral blood vessels leads to progressive, painful, necrotic skin ulcers resulting from occlusion of microvessels, due to deposition of calcium in the subcutaneous adipose tissue and dermis^3^. CVC is also a unique feature of coronary artery disease (CAD) and peripheral artery disease (PAD) in end stage kidney disease (ESKD). Medial calcification in these patients is associated with vascular stiffening and arteriosclerosis^4^. The most common type of medial calcification, Monckeberg’s sclerosis, occurs with greater frequency in patients undergoing dialysis than in those who are not undergoing dialysis^4^. While intimal and medial calcification may each occur independently, patients undergoing dialysis often exhibit both types^5^. Numerous complex mechanisms for cardiovascular calcification have been proposed^6^. Hyperphosphatemia, hypercalcemia, hyperparathyroidism, and abnormal vitamin D metabolism are known risk factors^7^, but the final common pathway is the deposition of hydroxyapatite crystals containing calcium, phosphate, and hydroxide [Ca_10_(PO_4_)_6_(OH)_2_]^6,8^.

There are currently no approved therapies to prevent or treat the progression of CVC and CVC related diseases like calciphylaxis or PAD in patients with ESKD. Supportive therapies include phosphate binders to reduce hyperphosphatemia^6^ and calcimimetics to address hyperparathyroidism^9^, thereby reducing hypercalcemia. However, these treatments do not directly target the formation and growth of hydroxyapatite crystals. SNF472, a selective calcification inhibitor, is in development to address these unmet medical needs in patients with ESKD on dialysis. SNF472 is an intravenous formulation of *myo*-inositol hexaphosphate (IP6 or phytate), which directly inhibits hydroxyapatite crystallization by binding selectively to the surface of the crystal. By inhibiting the final common pathway, the formation and growth of hydroxyapatite crystals, the efficacy of SNF472 is independent of the etiology of vascular calcification^10,11^. In vitro studies showed that at a concentration of 30 mg/L (45.5 μM) or above, SNF472 concentrations increase in blood while infused, reach a plateau, and remain nearly constant during dialysis^12^. Phase 1 and 2a clinical trials also showed that SNF472 was not dialyzable when it was infused during the dialysis session at doses ranging from 3 to 20 mg/kg^13,14^.

As described in other publications^15,16^, previously available tests for calcification, such as the T50 test to follow the transition of primary calciprotein particles (CPP) to secondary CPP or the determination of the levels of circulating fetuin-A-containing CPP might not be adequate to evaluate the effectiveness of potential therapeutic agents such SNF472, which act directly on the hydroxyapatite crystal at the vessel walls. Therefore, as a translational step from basic science to clinical development, we prepared and validated a pharmacodynamic (PD) biomarker assay to measure inhibition of calcium phosphate crystallization in plasma samples^15^. Results from plasma samples in rats and humans (with or without dialysis) validated the assay to measure inhibition of crystallization in the presence of polyphosphates, fetuin-A, sodium thiosulfate, and SNF472^15^. A phase 1b trial of SNF472 in patients undergoing dialysis provided further evidence supporting the ability of the assay to detect inhibition of calcium phosphate crystallization^14^.

A single-arm, open-label phase 2 trial to evaluate efficacy and safety in 14 patients with calciphylaxis showed improvement in wound healing and pain during 12 weeks of SNF472 treatment at a dose of approximately 7 mg/kg^17^. In a double-blind, placebo-controlled randomized phase 2b trial in patients on hemodialysis, SNF472 treatment for 52 weeks significantly attenuated the progression of coronary artery calcium and aortic valve calcification^18^. In this report, we evaluated the correlation between the PD assay and cardiovascular (heart and aorta) calcification in rats and the association between the PD assay and efficacy measures in patients who participated in these clinical trials.

## Results

### Animal study

**Figure 1:**
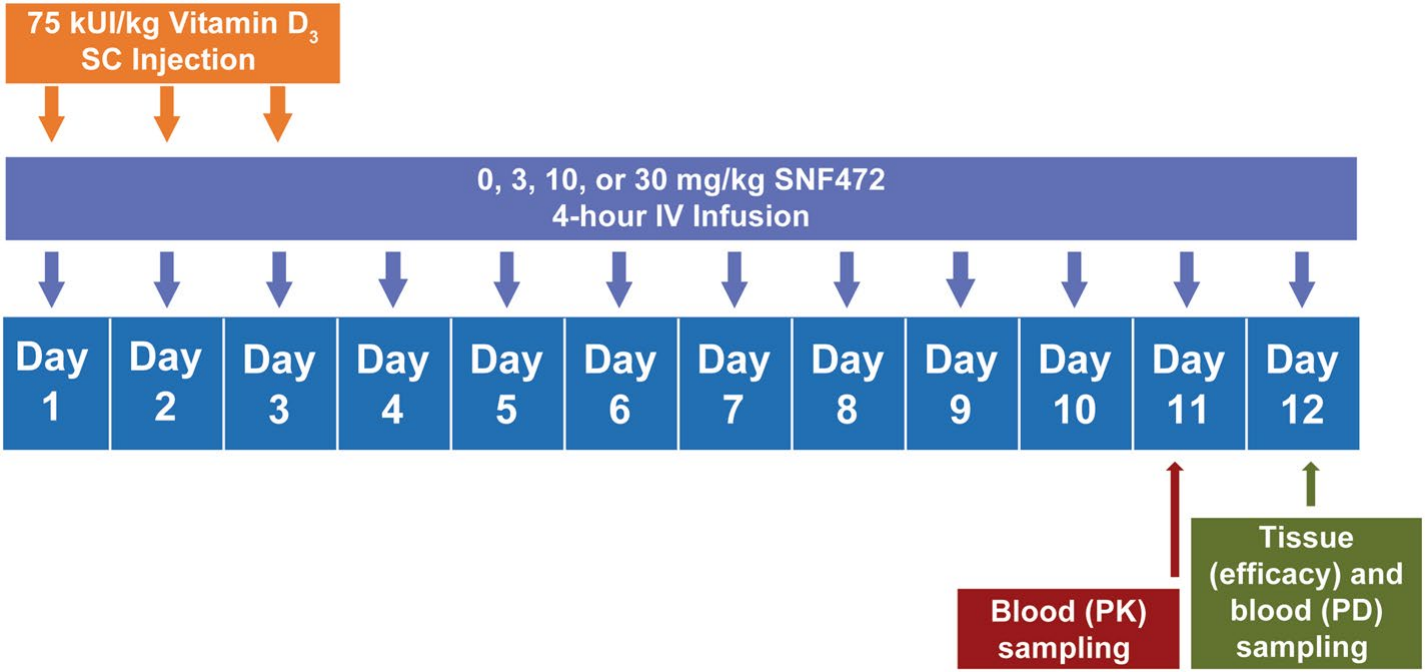
Animal study: experimental design. N = 10 for 0 mg/kg (saline) and 30 mg/kg groups; N = 9 for 3 and 10 mg/kg groups. IV = intravenous; SC = subcutaneous; PD = pharmacodynamics; PK = pharmacokinetics.

Rats (N = 38) received vitamin D from day 1 to day 3. Each rat received saline (n = 10) or SNF472 at 3 mg/kg (n = 9), 10 mg/kg (n = 9), or 30 mg/kg (n = 10) over a 4-h infusion period once daily from day 1 to day 12 (Fig. 1). Five animals died before day 12 in the 10 mg/kg (n = 1), 30 mg/kg (n = 3), and saline (n = 1) groups. We attributed the causes of death to vitamin D intoxication.

**Figure 2:**
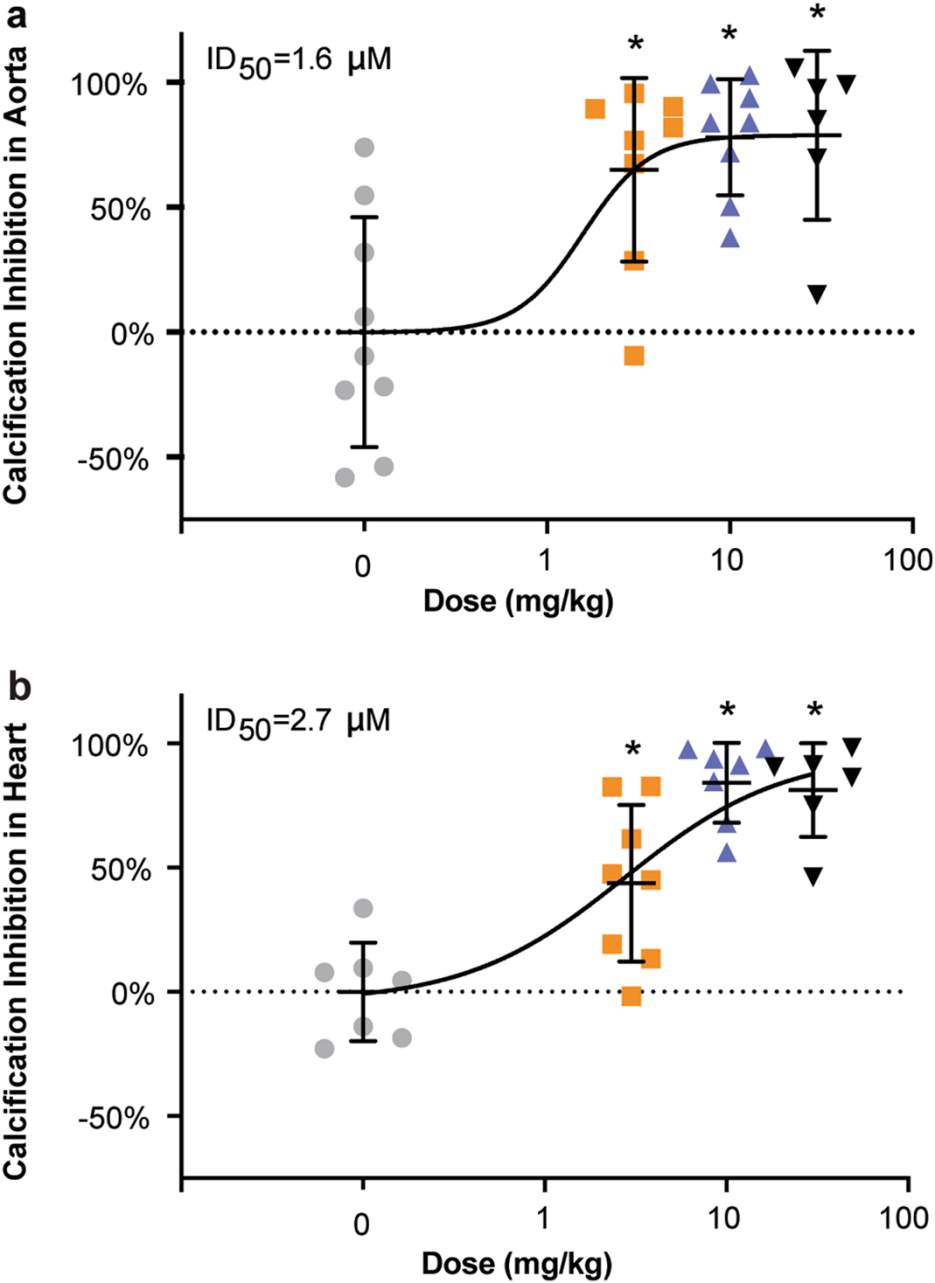
Inhibition of cardiovascular calcification in (**a**) aorta and (**b**) heart with SNF472 treatment in rats. Calcification was induced with daily subcutaneous injections of vitamin D (75,000 IU/kg) on day 1 to day 3. Rats were treated with 4-h intravenous infusions of saline (0 mg/kg) or SNF472 (3, 10, or 30 mg/kg) daily on day 1 to day 12. On day 12, animals were anesthetized, and the aorta and heart were excised for examination. Results are shown as percent inhibition of cardiovascular calcification relative to the control group that received saline infusions. Values are plotted as the mean, standard deviation, and individual points. Outlier results are excluded for two aortas (one each in the 3 mg/kg and 30 mg/kg groups) and five hearts (one in each SNF472 dosing group and two in the saline group). *P < 0.05 versus saline by one-way ANOVA.

Vitamin D injections for 3 days induced cardiovascular calcification in the heart and aorta. Administering SNF472 at 3 mg/kg for 12 days significantly (*P* < 0.05 vs saline) inhibited cardiovascular calcification by 65% in the aorta (Fig. 2a) and by 44% in the heart (Fig. 2b). Administering the 10 mg/kg and 30 mg/kg doses of SNF472 for 12 days achieved statistically significant (*P* < 0.05 vs saline), maximum inhibition of calcification (approximately 80%) in both the aorta and heart. Using a non-linear regression model, the mean dose of SNF472 required for 50% inhibition of calcification (ID_50_) was 1.6 mg/kg for the aorta and 2.7 mg/kg for the heart.

**Figure 3:**
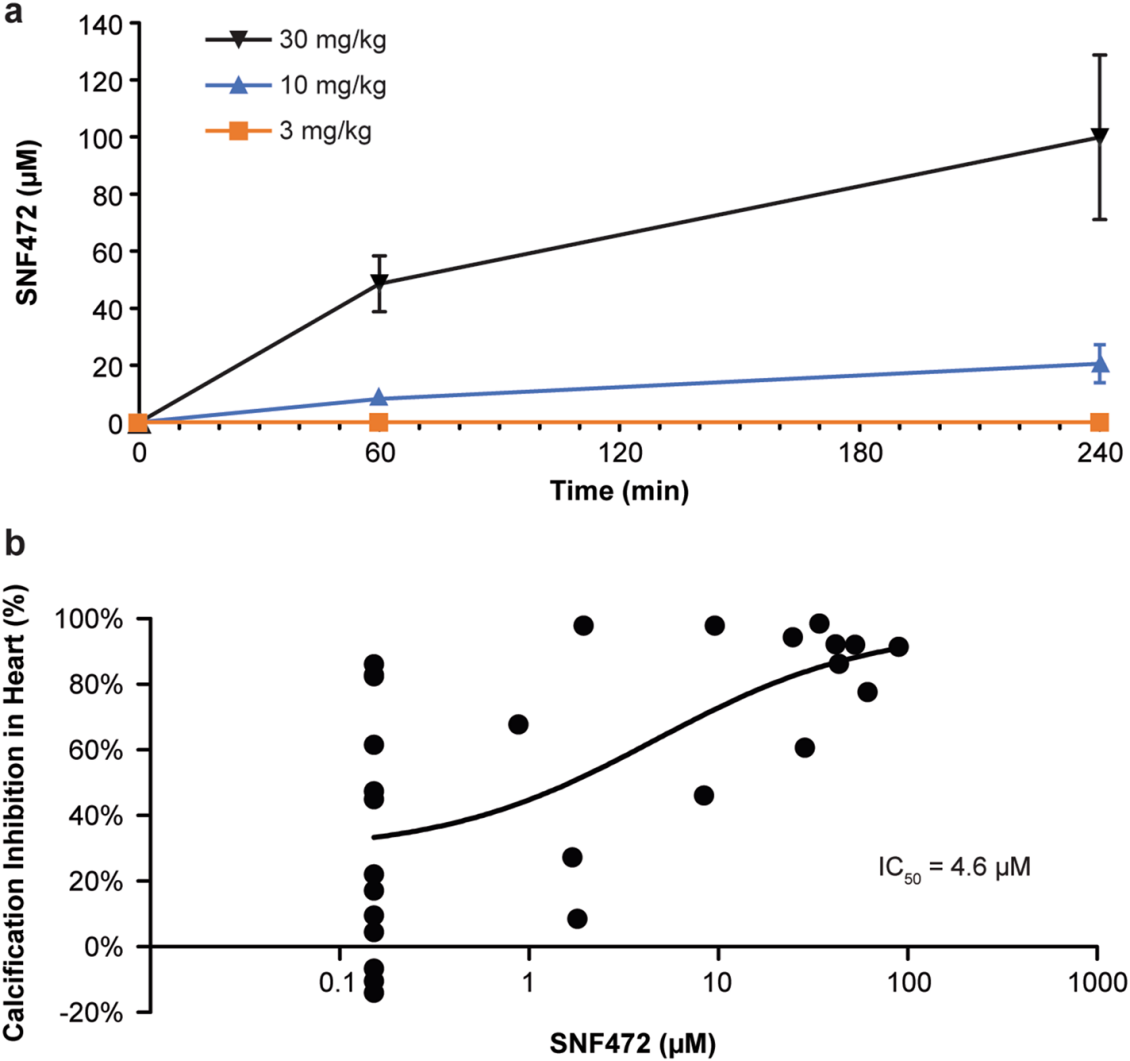
(**a**) Pharmacokinetics of SNF472 in rats treated with vitamin D to induce cardiovascular calcification. (**b**) Non-linear regression between calcification inhibition in heart and SN472 plasma levels in rats. The mean concentration of SNF472 required for 50% inhibition of calcification (IC_50_) was 4.6 µM for the heart and was not calculable for the aorta because of the high activity (65%) at the lowest dose of SNF472 tested. Calcification was induced with daily subcutaneous injections of vitamin D (75,000 IU/kg) on day 1 to day 3. Rats were treated with 4-h intravenous infusions of saline or SNF472 (3, 10, or 30 mg/kg) daily on day 1 to day 12. Plasma samples for SNF472 concentrations (mean ± SE) were obtained on day 11 of treatment with saline or SNF472. Plasma samples with concentrations below 1.5 µM (due to problems in the batch analysis) were not considered to calculate arithmetic mean. Plasma samples with concentrations below the limit of quantification (0.76 µM) were considered as zero to calculate the arithmetic mean.

Pharmacokinetic (PK) analysis at steady state on day 11 showed that SNF472 concentrations corresponded to the administered dose (Fig. 3a). SNF472 plasma concentrations were below the limit of quantification (LLOQ) of the validated bioanalytical method (0.76 µM) in rats receiving 3 mg/kg and dose-dependent in rats receiving 10 or 30 mg/kg. Mean C_max_ (the concentration at the end of the 4-h infusion) was 21 µM for a dose of 10 mg/kg and 100 µM for a dose of 30 mg/kg. Using a non-linear regression model, the mean concentration of SNF472 required for 50% inhibition of calcification (IC_50_) was 4.8 µM for the heart (Fig. 3b). The IC_50_ was not calculable for the aorta because of the high activity (65% inhibition of calcification) at the lowest dose of SNF472 tested.

**Figure 4:**
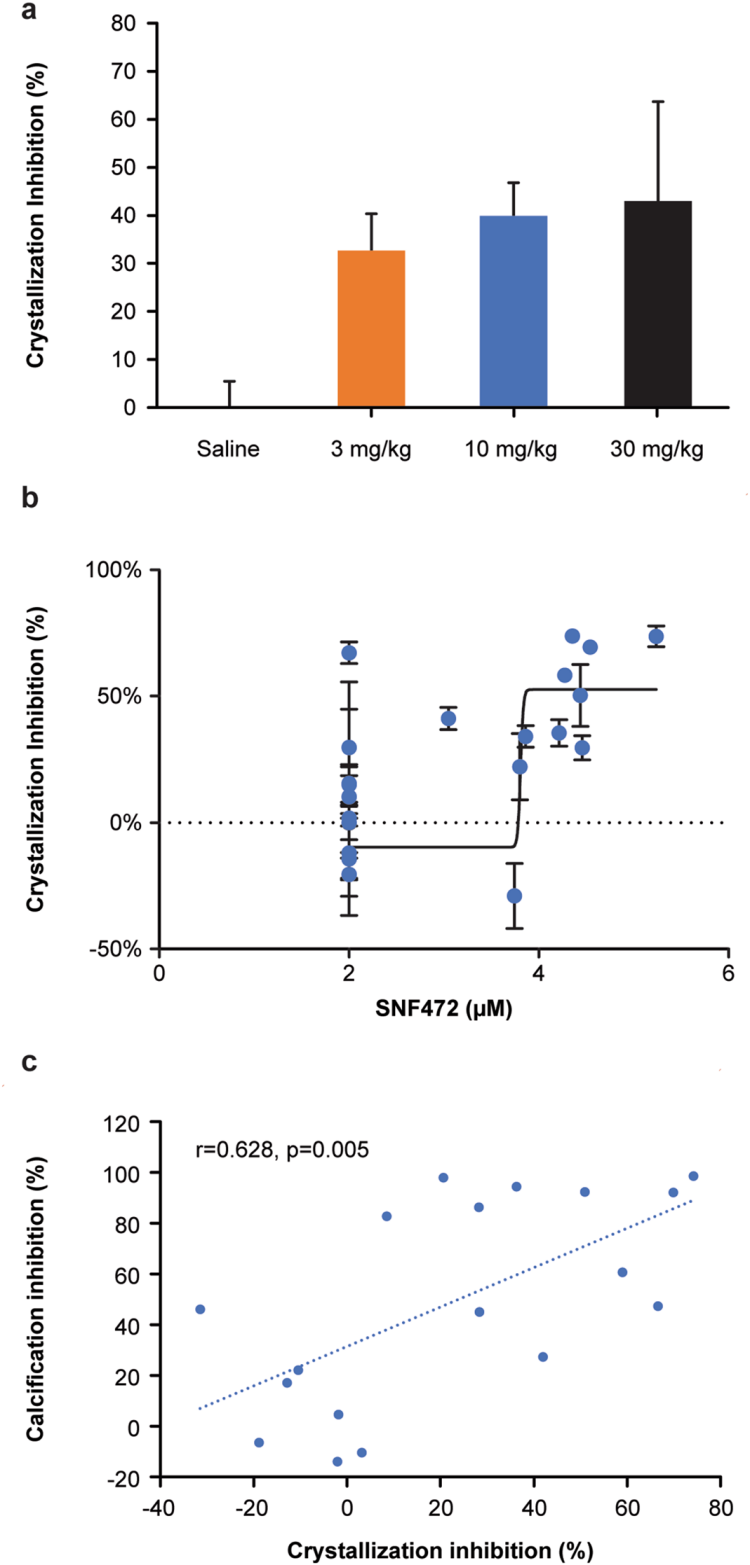
Results for inhibition of plasma crystallization and cardiovascular calcification in rats treated with vitamin D and SNF472. (**a**) Percent inhibition of plasma calcium phosphate crystallization by dose of SNF472 (median, interquartile range, and individual points). (**b**) Relationship between SNF472 circulating levels and inhibition of plasma calcium phosphate crystallization. Mean (± SEM) of 6 replicates assayed per sample. (**c**) Correlation between inhibition of cardiovascular calcification and inhibition of plasma calcium phosphate crystallization. To measure calcification, rats were treated with daily subcutaneous injections of vitamin D (75,000 IU/kg) on day 1 to day 3. To measure pharmacodynamic effects, rats were treated with 4-h intravenous infusions of saline or SNF472 (3, 10, or 30 mg/kg) daily on day 1 to day 12, then plasma calcium phosphate crystallization was induced ex vivo on day 12 by adding ionized calcium (12.5 mM) and phosphate (1.5 mM) to plasma samples, followed by spectrophotometric measurements at 550 nm every 3 min for 30 min.

Using the PD assay, SNF472 significantly and dose-dependently inhibited the formation of calcium phosphate crystals (Fig. 4a). Using a non-linear regression model, inhibition of calcium phosphate crystallization had a concentration–response relationship with SNF472 plasma levels at C_max_, showing an IC_50_ of 17 μM and an IC_80_ of 30 µM (Fig. 4b). We observed a direct correlation (r = 0.628, *P* = 0.005) between the PD assay results and the inhibition of cardiovascular calcification (Fig. 4c).

### Calciphylaxis clinical trial

The phase 2 open-label, single-arm clinical trial enrolled 14 patients with calciphylaxis lesions, with 11 patients completing 12 weeks of SNF472 treatment, at a dose of approximately 7 mg/kg, infused during each dialysis session. Efficacy and safety results from this study were reported previously^17^.

One patient’s plasma concentration of SNF472 was below the LLOQ (0.76 µM) at the end of the last infusion for unknown reasons. This patient’s SNF472 concentration at the end of the first infusion was similar to the values observed for the other patients in the study. PK and PD results for this patient after the last infusion were omitted from the summary statistics. The mean C_max_ at the end of the first (week 1 day 1) and last (week 12 day 5) infusions were 29 µM and 28 µM, respectively (Table 1). Median C_max_ at the end of the first and last infusions was 24 µM (range 3–62) and 21 µM (range 4–61), respectively. The accumulation factor (Rac C_max_) ranged from 0.2 to 2.5, with a mean value of 1.3 and a coefficient of variation of 69%.

**Table 1:** Summary of pharmacokinetic and pharmacodynamic results at the end of the first and last infusion of SNF472 during hemodialysis in the phase 2 clinical trial of patients with calciphylaxis.

Patient	Week 1 day 1		Week 12 Day 5		Rac C_max_	∆C_max_ (µM)^c^	∆PD effect (%)^c^
	Plasma C_max_^a^ (µM)	PD effect (%)^b^	Plasma C_max_^a^ (µM)	PD effect (%)^b^			
1	14.5	65.1	23.9	86.3	1.6	9.4	21.2
2	2.8	66.9	6.8	65.4	2.5	4.1	-1.5
3	29.0	38.7	NA	NA	NQ	NQ	NQ
4	21.0	79.5	4.3	59.0	0.2	-16.7	-20.5
5	36. 8	51.0	NA	NA	NQ	NQ	NQ
6	9.4	61.7	20.7	75.7	2.2	11.3	14.0
7	15.8	74.5	< LLOQ^d^	− 15.0^d^	NQ	NQ	NQ
8	12.9	46.7	NA	NA	NQ	NQ	NQ
9	61. 6	80.2	18.8	49.0	0.3	-42.7	-31.2
10	22.1	78.6	48.5	78.8	2.2	26.4	0.2
11	62.4	77.0	15.2	67.7	0.2	-47.3	-9.3
12	42.9	45.2	60.9	56.9	1.4	18.0	11.7
13	25.9	72.2	NA	NA	NQ	NQ	NQ
14	50.8	71.0	54.1	76.4	1.1 s	3.3	5.4
N	14	14	9	9	9		
Mean	29	65	28	68	1.3		
SD	19	14	21	12	0.9		
CV (%)	66	22	75	18	69		
Median	24	69	21	68	1.4		
Min–Max	3–62	39–80	4–61	49–86	0.2–2.5		

< LLOQ, below limit of quantification (0.76 µM); NA, sample not available; C_max_, maximum concentration; CV, coefficient of variation; NQ, not quantifiable; PD, pharmacodynamic; Rac C_max_, accumulation factor calculated as C_max,week12day5_/C_max,week1day1_.

^a^Plasma concentration obtained at the end of infusion.

^b^PD effect = –[inhibition].

^c^Individual difference between values obtained in week 12 day 5 and week 1 day 1.

^d^Values not considered in summary statistics because plasma concentration was below the limit of quantification at the end of infusion.

**Figure 5:**
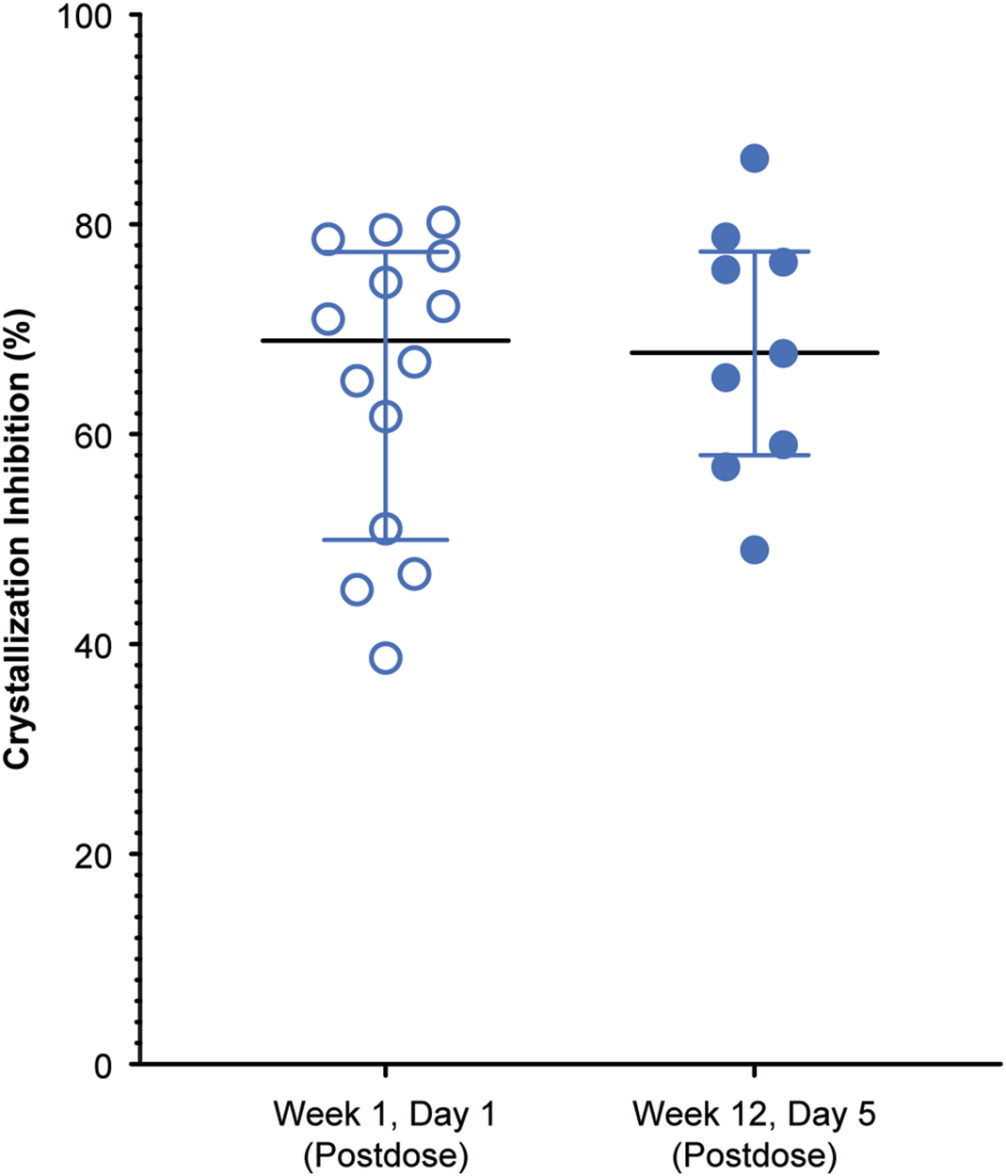
Pharmacodynamic results in the phase 2 clinical trial of SNF472 treatment during hemodialysis in patients with calciphylaxis. Values are plotted as the median, interquartile range, and individual points. Patients with calciphylaxis undergoing hemodialysis (N = 14) received SNF472 at a weight-based dose corresponding to 7 mg/kg during dialysis sessions for 12 weeks. Crystallization inhibition was measured at the end of the infusion for the first dose (week 1 day 1) and the last dose (week 12 day 5). One value for the last dose was not considered in summary statistics because the patient’s plasma concentration was below the limit of quantification at the end of infusion.

For the PD assay, the crystallization inhibition values were similar on the first and last days of SNF472 administration, with a mean of 65% (median, 69%; range 39–80%) after the first infusion and a mean of 68% (median, 68%; range 49–86%) after the last infusion (Fig. 5 and Table 1). Thus, inhibition of calcium phosphate crystallization in plasma was consistent across 12 weeks of SNF472 treatment and was in the plateau phase of the previously described PD effect of this compound^13,14,17^.

**Figure 6:**
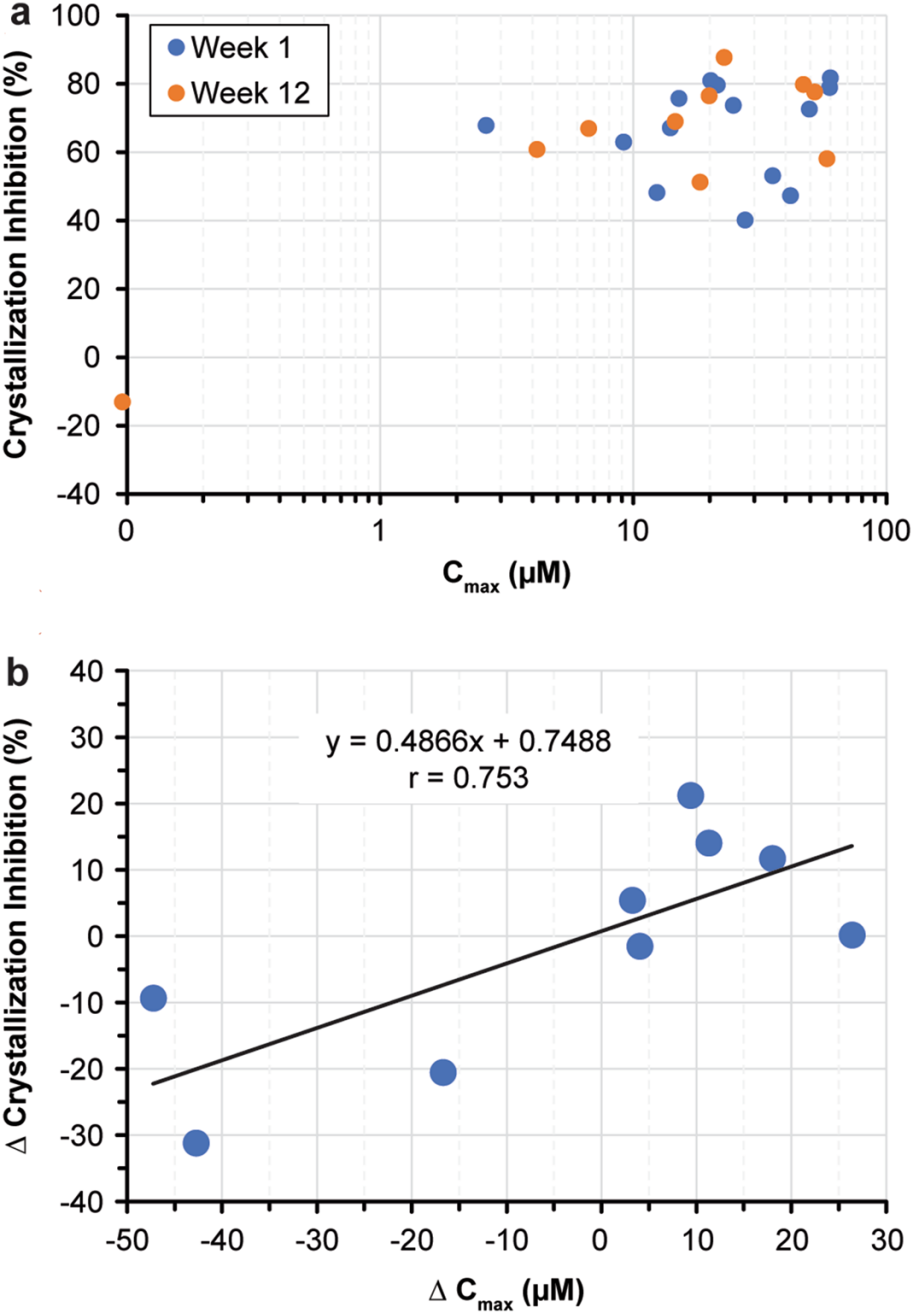
(**a**) Pharmacokinetic-pharmacodynamic relationship for SNF472. (**b**) Individual changes of the pharmacodynamic effect with plasma concentrations of SNF472 (week 12 vs. week 1). Patients undergoing hemodialysis with calciphylaxis (N = 14) received SNF472 at a weight-based dose corresponding to 7 mg/kg during dialysis for 12 weeks. Plasma crystallization inhibition was measured at the end of the infusion for the first dose (week 1 day 1) and the last dose (week 12 day 5). Plasma samples with concentrations below the limit of quantification (0.76 µM) were considered zero.

Because the SNF472 plasma concentrations were higher than the anticipated PD plateau, the PK-PD relationship could not be clearly established (Fig. 6a). Greater than 50% inhibition of calcium phosphate crystallization was observed in 83% (19 of 23) of end-of-infusion measurements at either visit, with median SNF472 concentrations of 22 µM (3–62 µM). At week 12 day 5, 8 of 9 patients with end-of-infusion measurements had greater than 50% inhibition of calcium phosphate crystallization with median SNF472 end-of-infusion concentrations of 21 µM (4–61 µM). For individual patients, the change in PD effect from week 1 to week 12 showed a positive correlation (r = 0.753) with the change in SNF472 plasma concentrations during the same interval (Fig. 6b).

The relationship between PD results and efficacy measures (changes in wound scores or in pain scores) could not be explored in this trial due to several limitations: only one dose level was used in a limited number of patients, there was no placebo group, and plasma concentrations were above the plateau of the PD assay for most of the samples.

### Cardiovascular calcification clinical trial (CaLIPSO Study)

The phase 2b randomized, double-blind, placebo-controlled clinical trial enrolled 274 patients with coronary artery calcification and assigned them to 52 weeks of treatment with placebo (n = 91), SNF472 300 mg (n = 92), or SNF472 600 mg (n = 91). CT Scans to determine calcium volume scores were conducted at baseline and week 52. Efficacy and safety results from this study were reported previously^18^.

**Figure 7:**
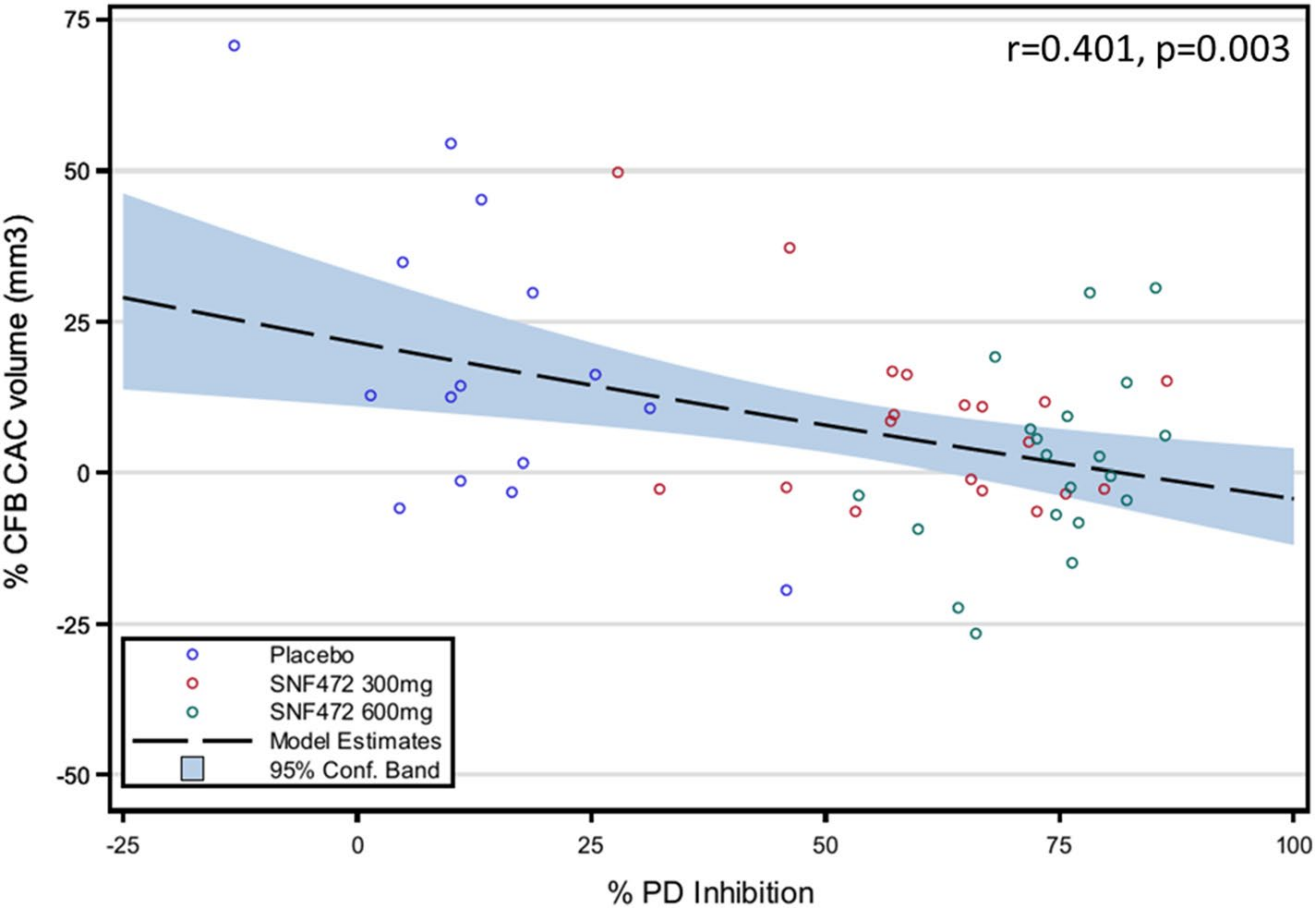
Linear model for the relationship of % change from baseline in coronary artery calcium (CAC) volume at week 52 versus average PD effect (%). Patients undergoing hemodialysis (N = 55) received placebo or SNF472 at doses of 300 or 600 mg/kg during dialysis, thrice weekly, for 52 weeks. Plasma crystallization inhibition was measured at the end of the infusion at weeks 1, 10, 22 and 52, and the average % inhibition of calcium phosphate crystallization found in these 4 sampling times was calculated and used in the model, as a read-out for the entire treatment of 52 weeks.

The results of the PD assay for inhibition of calcium phosphate crystallization across the visits at week 1, 10, 22, and 52 were 15% ± 17% for placebo, 61% ± 19% for SNF472 300 mg, and 75% ± 9% for SNF472 600 mg (mean ± SD). There was an inverse correlation between the results of the PD assay and the primary efficacy endpoint (percent change from baseline to Week 52 in the coronary artery calcification score by volume [CAC volume]). Patients with greater inhibition of calcium phosphate crystallization had lower progression of CAC. A linear model fitted with the experimental data showed r = 0.401 with *P* = 0.003 and (Fig. 7). The estimated percent change from baseline to week 52 in CAC volume had an X-axis intercept of 22% (95% CI 11% to 33%) and slope of -0.238 (95% CI -0.389 to 0.086). Interestingly, the X-axis intercept for no inhibition of crystallization in this analysis coincided with the observed progression rate in CAC volume for the placebo group in the CaLIPSO study using either the modified intent-to-treat population (20%) or the per protocol population (24%)^18^.

## Discussion

Increased CVC in patients with ESKD undergoing dialysis is associated with higher morbidity and mortality than the general population^19^. Therefore, targeting vascular calcification directly could have a beneficial role in CVC-related diseases including calciphylaxis, PAD, and coronary disease. However, clinical trials to assess the potential effects of anti-calcification compounds on CVC, and how this translates to decreased risk of events, require a long period of study. Therefore, new biomarkers are needed to assess the potential activity of new inhibitors of calcification.

We previously described our development and validation of a PD assay to measure calcium phosphate crystallization by spectrophotometry under standard temperature and agitation conditions that simulate crystallization^15^. In vitro, the PD assay showed concentration-dependent inhibition of calcium phosphate crystallization by up to 80% with addition of SNF472 to control rat plasma. In healthy rats, subcutaneous administration of SNF472 inhibited calcium phosphate crystallization in blood with a steep dose–response relationship and a narrow range between initial evidence of activity and asymptotic maximal inhibition of up to 70%.

In a phase 1b clinical study, we previously used the PD assay to show that infusion of SNF472 during hemodialysis dose-dependently inhibited calcium phosphate crystallization^14^. In that study, one cohort of patients received SNF472 doses ranging from 3 to 20 mg/kg for 1 week, and another cohort received SNF472 at a fixed dose of 10 mg/kg for 4 weeks. Mean ID_50_ and ID_80_ for SNF472 (doses associated with 50% and 80% inhibition of calcium phosphate crystallization) were 2.2 and 5.6 mg/kg, respectively.

Each of our previous studies examined the effects of SNF472 on calcium phosphate crystallization in healthy animals or in human volunteers without calcification disorders at study entry. In this paper, we showed that effects of SNF472 on the PD assay correlated with reduction in progression of pre-existing CVC. In rats, we administered vitamin D to induce CVC and showed significant inhibition of plasma calcium phosphate crystallization and heart calcification in a dose-dependent manner when we administered SNF472. Results obtained in the PD assay correlated directly with inhibition of calcification in the heart.

We then applied the PD assay in an open-label phase 2 trial, which showed that SNF472 treatment for 12 weeks in 14 patients with calciphylaxis on hemodialysis improved wound healing and pain^17^. In this study, the PD assay showed that SNF472 inhibited calcium phosphate crystallization by nearly 70% from the start to the end of treatment. The study had no placebo group and the PD effect reached the plateau of the dose–response. Therefore, a correlation between PD effect and efficacy measures could not be established in the calciphylaxis clinical trial.

We overcame this limitation when we applied the PD assay again in a phase 2b, randomized, placebo-controlled trial evaluating the efficacy and safety of SNF472 in 274 patients with CVC on hemodialysis. The primary report from the CVC study showed that SNF472 administered for 52 weeks was well tolerated and significantly inhibited progression of CVC^18^. The PD assay showed at least 50% inhibition of calcium phosphate crystallization for most of the patients who received SNF472. The inclusion of a placebo group and two SNF472 treatment groups (300 mg and 600 mg) allowed us to build a model showing a linear relationship between the PD assay effect and a decrease in CVC progression over 52 weeks in humans^18^. The correlation between PD effect and inhibition of CVC (progression of CAC volume) in this clinical trial was similar to the correlation that we observed in our animal models.

In conclusion, in this report we used our previously validated PD assay for calcium phosphate crystallization to show that SNF472 substantially and consistently inhibits calcium phosphate crystallization with repeated dosing, and that this PD effect correlates with reduction in CVC progression in rats and in clinical effect in patients with ESKD on hemodialysis. This translational, noninvasive tool may be used in future research to assess the potential therapeutic effects of calcification inhibitors such as SNF472 in CVC and related diseases like calciphylaxis, CAD, or PAD.

## Methods

### PD assay

In a previous report we described the development and validation of the PD assay for inhibition of calcium phosphate crystallization^15^. For this assay, we added ionized calcium 12.5 mM and phosphate 1.5 mM to plasma samples ex vivo to induce calcium phosphate crystallization, and agitated the samples at 750 rpm for 30 min. We obtained spectrophotometric measurements at 550 nm every 3 min and plotted the increase in absorbance versus the logarithm of time to calculate the slope for the linear interval. Each plasma sample generated one value for the slope. We calculated inhibition of calcification induction as the percentage change in the slope for the linear interval between pre-dose and post-dose (end of infusion) samples Eq. (1):

1 $$ \% \,{\text{change}}\,({\text{inhibition}}) = [( {\text{slope}}\,{\text{post-dose}}-{\text{slope}}\,{\text{pre-dose}}) \div {\text{slope}}\,{\text{pre-dose}}] \times 100. $$

### Animal study

Animals were kept at constant temperature, humidity, and on a 12-h light/dark cycle with free access to water and rat chow. The study was approved by and conducted according to the guidelines of the local animal ethics committee (Comitè ètic d’experimentació animal, Catalunya, Spain; Decree 214/97). The experimental design for the animal study is summarized in Fig. 1. We divided 38 Sprague Dawley rats into four groups of 9 or 10 animals each. We implanted an intravenous polyurethane catheter (0.62 × 1.02 mm, Instech Laboratories, Plymouth Meeting, PA, USA) into the jugular vein of each animal during the acclimatization period, which lasted 6 to 8 days before the start of treatment and calcification induction. We externalized the catheter through an incision in the interscapular region and attached it to a delivery system via a tether system with a harness and a swivel joint (Instech Laboratories). We connected this system to an 8-channel infusion pump (New Era 1800, Farmingdale, NY, USA). Each day we filled the catheter with fresh heparinized saline to avoid coagulation.

We induced cardiovascular calcification in all rats by administering vitamin D 75,000 IU/kg subcutaneously daily from day 1 to day 3. In the 4 groups, we administered saline or SNF472 at 3, 10, 30 mg/kg daily from day 1 to day 12 by 4-h intravenous infusion. We weighed each animal daily.

On day 11, before infusion and 60 and 240 min after the infusion start, we collected approximately 200 μL of blood from each animal in a K_2_EDTA vacuette tube without interrupting the infusion. We quantified SNF472 levels in blood by liquid chromatography-mass spectroscopy^20^.

On day 12, we anesthetized each animal with isoflurane at the end of the 4-h infusion and performed cardiac puncture to extract total blood (8–10 mL) into collection tubes for plasma (K_3_EDTA, 6 mL) and serum (2–3 mL). We used the PD assay to measure calcium phosphate crystallization in the plasma samples. After exsanguination, we sacrificed the animals and performed necropsy, collecting the aorta, heart, right kidney, and femurs for calcium content measurement by inductively coupled plasma optical emission spectrometry after acidic digestion^21^.

We used a boxplot assay to detect outliers, which we discarded from the statistical analysis, and summarized the remaining results by treatment group. Outliers were defined as the observations that were below first quartile minus 3 times the Interquartile range (IQR) or above third quartile plus 3 times the IQR. In each group, we calculated the mean ID_50_ and the mean IC_50_ in the heart and aorta using a non-linear fitting program (GraphPad Prism; GraphPad Software, La Jolla, CA, USA) and semi-logarithmic concentration response curves. We performed a one-way ANOVA to detect the effect on tissue calcification of each dose of SNF472 compared with saline. When significant effects were observed (*P* < 0.05 vs saline), we applied a post-hoc least significant difference test to identify differences between groups. For PD analysis, we performed a Kruskal–Wallis analysis for non-normally distributed data. We conducted a bivariate Pearson correlation test between PD assay results and cardiovascular calcification results and a four-parameter variable slope non-linear regression fixing the top at 65.7% (maximum degree of inhibition obtained in the validation of the method in rat ex vivo samples^15^) between PD assay results and SNF472 plasma levels at C_max_.

### Calciphylaxis clinical trial

The design of the calciphylaxis clinical trial has been published in detail^17^. In this open-label, single-arm clinical trial, we added SNF472 to standard care for 12 weeks in 14 patients undergoing hemodialysis with calciphylaxis. This report includes the results of PK and PD assessments. Clinical results for safety and effectiveness (wound healing, pain, and wound-related quality of life), were reported separately^17^. Eligible patients were ≥ 18 years of age and had clinically diagnosed calciphylaxis, which could include either newly diagnosed calciphylaxis or recurrent calciphylaxis that had been dormant with no skin lesion involvement for at least 90 days before study start. We administered SNF472 intravenously three times per week for 2.5 to 4 h, during each dialysis session, using an SNF472 dose of 400, 450, 700 or 900 mg for patients with a dry body weight of 50 to < 66 kg, 66 to < 81 kg, 81 to < 111 kg, or 111 to 150 kg, respectively, to achieve a weight-based dose of approximately 7 mg/kg.

We collected blood samples (4 mL), using the dialysis tubing so the patient was not disturbed, into polyethylene terephthalate tubes containing potassium (K_3_EDTA) before hemodialysis and at the end of SNF472 infusion for the first dose (baseline; day 1 of week 1) and last dose (day 5 of week 12). We measured SNF472 concentrations with a validated liquid chromatography-mass spectroscopy method^20^. We used another aliquot of plasma from the blood sample for the PD assay to measure calcium phosphate crystallization.

Each patient had two values for the percentage change in calcium phosphate crystallization (ie, percent inhibition): one at week 1 and one at week 12. We compared percent inhibition at week 1 and week 12 using a Wilcoxon signed-rank test as a non-parametric statistical hypothesis test, with an alpha level of 0.05 to compare the two related samples. We used percent inhibition and end-of-infusion plasma levels (C_max_) to evaluate the potential PK-PD correlation. Available samples were included in the analysis for pharmacokinetic/pharmacodynamic correlation if the SNF472 concentration was above the limit of detection. A total of 23 samples met these criteria: 14 samples at week 1 day 1, and 9 samples at week 12 day 5.

Patients gave written informed consent to participate. An ethics committee for each site (New England Independent Review Board for centers in the USA and the North West—Liverpool Central Research Ethics Committee for centers in the UK) approved the trial design, which was conducted in accordance with the Declaration of Helsinki and was consistent with International Council for Harmonisation Guidelines on Good Clinical Practice and regulatory requirements. The clinical trial was registered at ClinicalTrials.gov as #NCT02790073.

### Cardiovascular calcification clinical trial (CaLIPSO Study)

The design of the cardiovascular calcification trial has been published in detail^18^. Briefly, we randomized 274 adult patients (18–80 years of age) on hemodialysis with a baseline CAC Agatston score of 100 to 3500 units on a screening non-contrast multi-detector computed tomography (MDCT) to receive placebo, SNF472 300 mg, or SNF472 600 mg, infused thrice weekly during hemodialysis for 52 weeks. MDCT imaging was repeated at week 52. Collection of blood samples at weeks 1, 10, 22, and 52, and treatment of blood samples to perform the PD assay were as described for the calciphylaxis clinical trial. A linear model was used to evaluate the relationship between PD and percent change from baseline to week 52 for CAC volume. This model is described by the formula:

$$ {\text{Y}} = {\text{b}}_{0} + {\text{b}}_{1} {\text{X}} +\upvarepsilon _{{\text{i}}} $$

where Y, value of the response; b_0_, intercept; b_1_, parameter estimate for variable X; ε_i_, random error term. A standard assumption, adopted here, is that the ε_i_ terms are independent identically distributed with a mean of 0 and variance σ^2^. An additional standard assumption is that the error terms are normally distributed.

Linear model fitting was summarized by the value of the adjusted coefficient of correlation (R).

The average PD value for each particular patient was calculated as the mean value of individual PD values found in weeks 1, 10, 22, and 52 of treatment included in the per protocol population.

The study was approved by the research ethics committee of each participating institution and the trial was conducted according to the principles of the Declaration of Helsinki. The clinical trial was registered at ClinicalTrials.gov as #NCT02966028.

## References

[1] Mizobuchi M, Towler D, Slatopolsky E (2009). Vascular calcification: the killer of patients with chronic kidney disease. J. Am. Soc. Nephrol..

[2] New SE, Aikawa E (2011). Cardiovascular calcification: an inflammatory disease. Circ. J..

[3] Nigwekar SU, Thadhani R, Brandenburg VM (2018). Calciphylaxis. N. Engl. J. Med..

[4] Lanzer P (2014). Medial vascular calcification revisited: review and perspectives. Eur. Heart J..

[5] Johnson RC, Leopold JA, Loscalzo J (2006). Vascular calcification: pathobiological mechanisms and clinical implications. Circ. Res..

[6] Giachelli CM (2004). Vascular calcification mechanisms. J. Am. Soc. Nephrol..

[7] Mazhar AR (2001). Risk factors and mortality associated with calciphylaxis in end-stage renal disease. Kidney Int..

[8] Joubert P, Ketteler M, Salcedo C, Perello J (2016). Hypothesis: Phytate is an important unrecognised nutrient and potential intravenous drug for preventing vascular calcification. Med Hypotheses.

[9] Savica V, Santoro D, Monardo P, Mallamace A, Bellinghieri G (2008). Sevelamer carbonate in the treatment of hyperphosphatemia in patients with chronic kidney disease on hemodialysis. Ther. Clin. Risk Manag..

[10] Grases F (2008). Phytate inhibits bovine pericardium calcification in vitro. Cardiovasc. Pathol..

[11] Grases F, Costa-Bauza A (1999). Phytate (IP6) is a powerful agent for preventing calcifications in biological fluids: usefulness in renal lithiasis treatment. Anticancer Res..

[12] Perelló J (2018). SNF472, a novel inhibitor of vascular calcification, could be administered during hemodialysis to attain potentially therapeutic phytate levels. J. Nephrol..

[13] Perelló J (2018). First-time-in-human randomized clinical trial in healthy volunteers and haemodialysis patients with SNF472, a novel inhibitor of vascular calcification. Br. J. Clin. Pharmacol..

[14] Salcedo C (2019). A phase 1b randomized, placebo-controlled clinical trial with SNF472 in haemodialysis patients. Br. J. Clin. Pharmacol..

[15] Ferrer MD (2017). A novel pharmacodynamic assay to evaluate the effects of crystallization inhibitors on calcium phosphate crystallization in human plasma. Sci. Rep..

[16] Pasch A (2016). Novel assessments of systemic calcification propensity. Curr. Opin. Nephrol. Hypertens.

[17] Brandenburg VM (2019). Improvement in wound healing, pain, and quality of life after 12 weeks of SNF472 treatment: a phase 2 open-label study of patients with calciphylaxis. J. Nephrol..

[18] Raggi P (2020). Slowing progression of cardiovascular calcification with SNF472 in patients on hemodialysis: results of a randomized Phase 2b study. Circulation.

[19] Schiffrin EL, Lipman ML, Mann JF (2007). Chronic kidney disease: effects on the cardiovascular system. Circulation.

[20] Tur F (2013). Validation of an LC-MS bioanalytical method for quantification of phytate levels in rat, dog and human plasma. J. Chromatogr. B Anal. Technol. Biomed. Life Sci..

[21] Ferrer MD (2018). Characterization of SNF472 pharmacokinetics and efficacy in uremic and non-uremic rats models of cardiovascular calcification. PLoS ONE.

