# Author Correction: A novel assay to measure calcification propensity: from laboratory to humans

**DOI:** 10.1038/s41598-021-83842-5

**Published:** 2021-02-16

**Authors:** M. Mar Perez, Miguel D. Ferrer, Marta Lazo-Rodriguez, Ana Zeralda Canals, Elisenda Banon-Maneus, Josep M. Campistol, Stephan Miller, Rekha Garg, Alex Gold, Carolina Salcedo, Joan Perelló

**Affiliations:** 1 Sanift Therapeutics, Parc Bit - Europa Building, 2nd Floor, 07121 Palma de Mallorca, Spain; 2 Department of Fundamental Biology and Health Sciences, University of the Balearic Islands, Palma, Spain; 3 Laboratori Experimental de Nefrologia I Trasplantament (LENIT), Fundació Clínic Per a La Recerca Biomèdica, Barcelona, Spain; 4 Spanish Kidney Research Network, ISCIII-RETIC, REDinREN RD016/0 009, Madrid, Spain; 5 Sanift Therapeutics, San Diego, CA USA; 6 Department of Medicine, Stanford University, Palo Alto, CA USA; 7 Laboratory of Renal Lithiasis Research, University of the Balearic Islands, Palma, Spain; 8 Present Address: PharmaDRS Consulting, LLC, San Diego, USA

Correction to: *Scientific Reports* https://doi.org/10.1038/s41598-020-74592-x, published online 16 October 2020

This Article contains errors in Figure 4, where an incorrect version of the figure file was published. The correct Figure 4 appears below as Figure 1.

**Figure 1:**
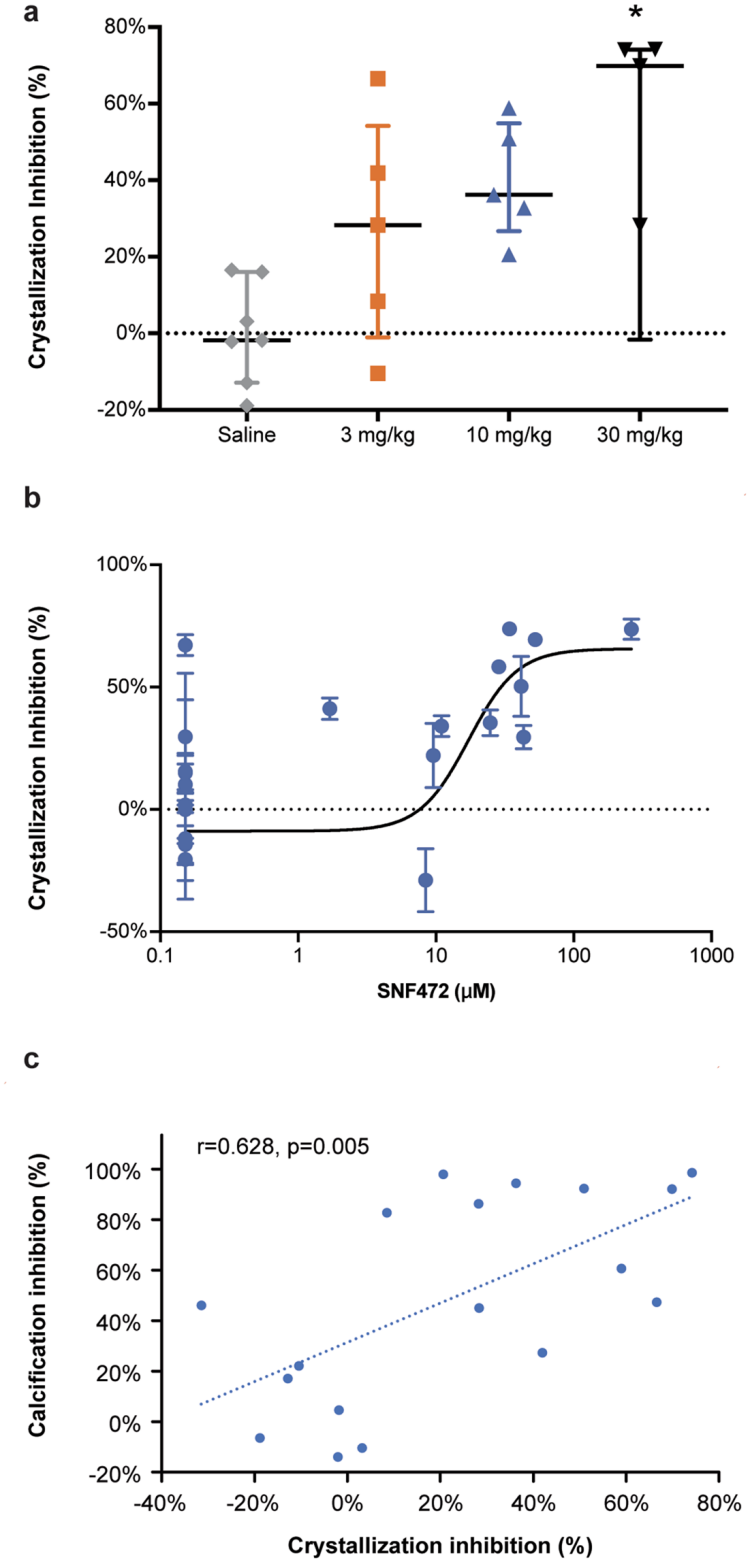
A correct version of the original Figure 4.

